# Blood transfusion and mortality in myocardial infarction: an updated meta-analysis

**DOI:** 10.18632/oncotarget.19208

**Published:** 2017-07-12

**Authors:** Zuomin Yin, Botao Yu, Weisheng Liu, Ketao Lan

**Affiliations:** ^1^ Department of Chest Pain Center, The Affiliated Central Hospital of Qingdao University, Qingdao, Shandong Province, China

**Keywords:** blood transfusion, myocardial infarction, mortality, meta-analysis

## Abstract

**Background:**

Several observational and preclinical studies have shown that blood transfusion may modify the mortality of patients with myocardial infarction (MI). The aim of this meta-analysis is to evaluate the recent evidence on the effectiveness of blood transfusion for all-cause mortality in patients with MI.

**Materials and Methods:**

PUBMED, EMBASE and the Cochrane central register of controlled trials were searched up to June 2016 by two independent investigators. Studies were considered eligible if they recruited adult MI patients and reported hazard ratio (HR) for all-cause mortality comparing those who received blood transfusion with those who did not receive blood transfusion. We abstracted and calculated pooled HRs using a random-effects model.

**Results:**

From 4277 unique reports, we identified 17 studies including 260811 patients with 11 studies examining short-term (in hospital/30-day) all-cause mortality and 9 studies examining long-term (more than 30 days) all-cause mortality. Meta-analysis demonstrated that patients treated with blood transfusion had increased short-term all-cause mortality (HR, 2.39, 95% CI 1.81 to 3.15) compared with those without blood transfusion treatment. Similar findings were observed by subgroup analyses. We also find significant association between blood transfusion and long-term all-cause mortality (HR 1.90, 95% CI 1.40 to 2.58) for MI patients.

**Conclusions:**

In patients with MI, blood transfusion treatment is associated with patient short-term and long-term all-cause mortality. However, further large-scale prospective studies are needed to establish its validity of this association.

## INTRODUCTION

Perioperative anemia is one of the commonest status for patients with acute myocardial infarction (MI) or other acute cardiovascular events, which always threats patient prognosis and is always associated with increased risk of all-cause mortality [1–3]. Blood transfusion is one of the most efficient ways to help MI patients improve myocardial oxygen delivery when red blood cells are transfused which may augment hemoglobin levels. Despite its recognized advantages of blood transfusion for MI, it may also increase some related risks, such as increased risk of infection, allergic reaction, high fever, volume overload and hemolytic reaction [4–6].

Though blood transfusions are widespread utilized in clinics, there still lack solid evidence whether this method will benefit patients with MI. Moreover, its safety and efficacy have not been strictly assessed by large clinical trials. Up till now, quite a few observational studies have reported short-term and long-term outcomes of blood transfusion for patients with MI with controversial findings [7–10]. Salisbury et al. used a large cohort of 34,937 acute MI hospitalizations from 57 centers and found that blood transfusion was significantly associated with reduced in-hospital mortality (hazard ratio [HR] 0.73 95% confidence interval [CI] 0.58 to 0.92) using propensity matching score analysis [11]. However, Ducrocq et al. did not find significant associations between transfusion and patient in hospital mortality as well as 5-year all-cause mortality by analyzing the data from the nationwide FAST-MI 2005 AMI registry [12].

Accordingly, we aimed to resolve the existing uncertainty regarding the prognostic effect of blood transfusion for MI patients by updating a systematic review and meta-analysis of all published data.

## RESULTS

### Study characteristics

**Figure 1:**
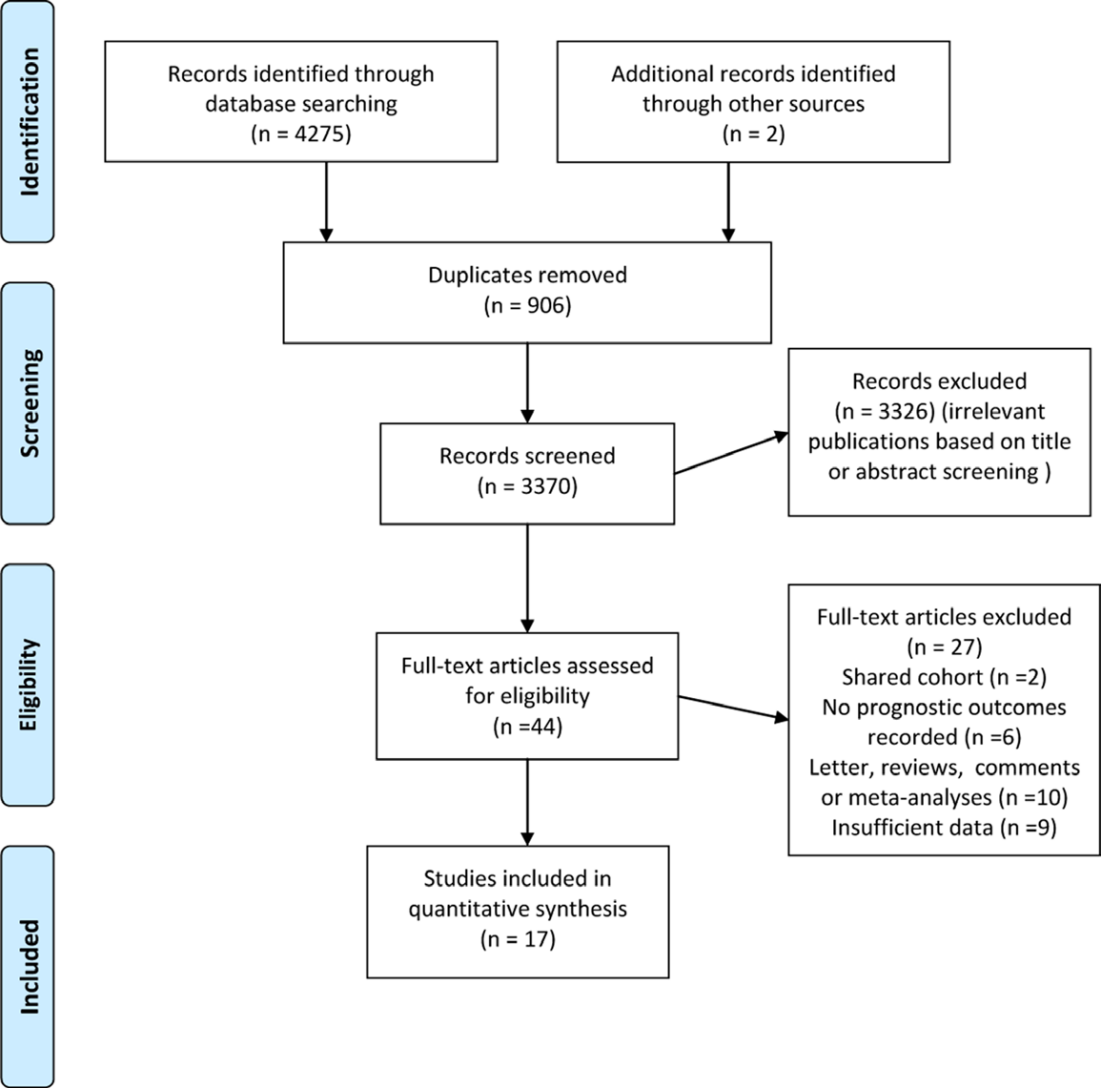
Flowdiagram of study selection process including the exclusion reasons

The search strategy revealed 4277 potentially suitable citations. After reading abstracts or titles, 44 relevant studies were selected for further full text review. As is shown in Figure 1, the reasons for study exclusion are presented. Finally, a total of 17 studies including 260811 patients were identified for analysis on the effectiveness of blood transfusion on all-cause mortality in patients with MI [8–24]. Among them, 11 studies reported short-term (in hospital/30-day) all-cause mortality and 9 studies examining long-term (more than 30 days) all-cause mortality. The clinical and methodological characteristics of each study are detailed in Supplementary Table 1 and Table 2. The majority (12/17) of the studies were conducted in USA [8–11, 17–24] and others were in Europe and Asia which were all published between 2001 and 2015. The median sample size of the included studies was 250 (range, 64 to 494), with a total of 815 patients received blood transfusion. Two studies used single center cohort and 13 involved multicentered cohorts. Five studies enrolled patients with ST-segment elevation myocardial infarction (STEMI), two with non-STEMI and the other ten with mixed type. The median baseline hemoglobin level of the patients was 11.25 g/dl (range 8.1 to 13.9). The follow-up duration ranged from 30 days to 5 years. Eight of 17 studies had a methodological quality score of more than seven (Table 2).

**Table 1A:** Subgroup analyses for short-term all-cause mortality of included studies according to baseline characteristics

	HR	95% CI	Heterogeneity (%)	*P*	No. of included Studies
**Total**	2.39	1.81 to 3.15	89.3		13
**Outcome**					
In hospital mortality	2.21	1.78 to 2.74	71.0	0.854	7
30-day mortality	2.40	1.22 to 4.74	92.3		6
**Region**					
US	2.30	1.68 to 3.14	91.3	0.525	10
Europe	2.30	0.88 to 6.03	75.2		2
**Single/Multicenter**					
Single	3.04	1.95 to 4.75	0	0.596	2
Multicenter	2.18	1.59 to 3.00	91.4		10
**Design**					
Retrospective	1.88	1.07 to 3.29	89.6	0.198	4
Prospective	2.73	2.03 to 3.68	81.2		9
**Sample size**					
≥ 3000	2.91	2.11 to 4.00	34.1	0.754	6
< 3000	2.09	1.44 to 3.05	94.8		7
**MI type**					
Non-STEMI + STEMI	2.09	1.38 to 3.16	90.7	0.095	8
Non-STEMI	1.83	1.29 to 2.70	47.4		2
STEMI	3.83	2.89 to 5.07	0		3
**Adjustement**					
Sufficient	2.28	1.64 to 3.16	92.2	0.791	9
Insufficient/unclear	2.79	1.62 to 4.80	60.5		4
**Study quality**					
≥ 8	1.97	1.35 to 2.86	92.3	0.259	8
< 8	3.11	2.30 to 4.20	54.4		5
**Publication type**					
Full text	2.33	1.70 to 3.20	90.7	0.668	11
Abstract	2.66	1.45 to 4.87	74.0		2
**Hemoglobin level (g/dl)**					
≤ 12	2.11	1.77 to 2.52	16.9	0.366	5
> 12	4.01	2.83 to 5.68	0		2
**Hematocrit (%)**					
> 33	3.18	1.74 to 5.79	16.5	0.953	4
≤ 33	3.14	1.98 to 4.98	95.6		3

Abbreviations: CI, confidence interval; HR, hazard risk.

**Table 1B:** Subgroup analyses for long-term all-cause mortality of included studies according to baseline characteristics

	HR	95%CI	Heterogeneity (%)	*P*	No. of included Studies
**Total**	1.90	1.40 to 2.58	80.9		9
**Outcome**					
30 d–1 y	2.10	1.61 to 2.74	0	0.267	3
≥1 y	1.82	1.18 to 2.79	87.2		6
**Region**					
US	2.25	1.40 to 3.63	81.4	0.660	4
Europe	1.48	0.53 to 4.12	89.4		2
Asian	1.90	1.27 to 2.84	-		1
**Single/Multicenter**					
Single	2.57	1.44 to 4.59	-	0.509	1
Multi	1.86	1.21 to 2.85	86.7		6
**Design**					
Retrospective	1.92	1.24 to 2.96	67.6	0.903	3
Prospective	1.89	1.22 to 2.92	86.0		6
**Sample size**					
≥ 3000	2.06	1.17 to 3.63	86.2	0.625	3
< 3000	1.82	1.24 to 2.68	79.0		6
Myocardial.In**farction.Types**					
non-STEMI;STEMI	1.82	1.04 to 3.19	85.5	0.816	4
STEMI	1.98	1.37 to 2.87	77.6		5
**Adjusted.variables**					
Sufficient	1.80	1.09 to 2.96	88.7	0.591	5
Insufficient/unclear	1.99	1.48 to 2.68	42.5		4
**Study quality**					
≥ 8	1.63	0.48 to 5.58	91.6	0.275	2
< 8	2.01	1.54 to 2.62	67.6		7
**Type**					
Fulltext	2.08	1.30 to 3.32	85.9	0.572	6
Abstract	1.60	1.19 to 2.17	51.5		3
**Hemoglobin**					
≤ 12	1.74	1.03 to 2.95	84.1	0.183	4
> 12	2.87	2.25 to 3.67	0		3
**Hematocrit**					
> 33	3.05	2.33 to 4.00	0	0.329	2
≤ 33	2.57	1.44 to 4.59	-		1

Abbreviations: CI, confidence interval; HR, hazard risk.

**Table 2:** Methodological quality of included studies based on the Newcastle–Ottawa Scale for included studies

Study	Selection	Comparability	Outcome/exposure	Overall quality (max 9)
Ducrocq (2015)	⋆⋆⋆⋆	⋆⋆	⋆⋆	8
Salisbury (2014)	⋆⋆⋆⋆	⋆⋆	⋆⋆	8
Tajstra (2013)	⋆⋆⋆	⋆	⋆⋆	6
Ergelen (2012)	⋆⋆⋆	⋆	⋆	5
Athar (2011)	⋆⋆⋆	⋆⋆	⋆⋆	7
Cooper (2011)	⋆⋆⋆	⋆⋆	⋆⋆⋆	8
Volenti (2010)	⋆⋆⋆	⋆⋆	⋆⋆	7
Cosgrove (2009)	⋆⋆⋆⋆	⋆	⋆⋆	7
Jolicœur (2009)	⋆⋆⋆⋆	⋆	⋆⋆	7
Nikolsky (2009)	⋆⋆⋆⋆	⋆⋆	⋆⋆	8
Shishehbor (2009)	⋆⋆⋆⋆	⋆	⋆⋆	7
Aronson (2008)	⋆⋆⋆	⋆	⋆⋆	6
Jani (2007)	⋆⋆⋆⋆	⋆	⋆⋆	7
Singla (2006)	⋆⋆⋆⋆	⋆⋆	⋆⋆	8
Yang (2005)	⋆⋆⋆⋆	⋆⋆	⋆⋆	8
Rao (2004)	⋆⋆⋆⋆	⋆⋆	⋆⋆⋆	9
WU (2001)	⋆⋆⋆⋆	⋆⋆	⋆⋆⋆	9

a Study quality assessment of observational studies performed using the Newcastle–Ottawa scale (each asterisk represents if individual criterion within the subsection were fulfilled).

### Short-term all-cause mortality

**Figure 2:**
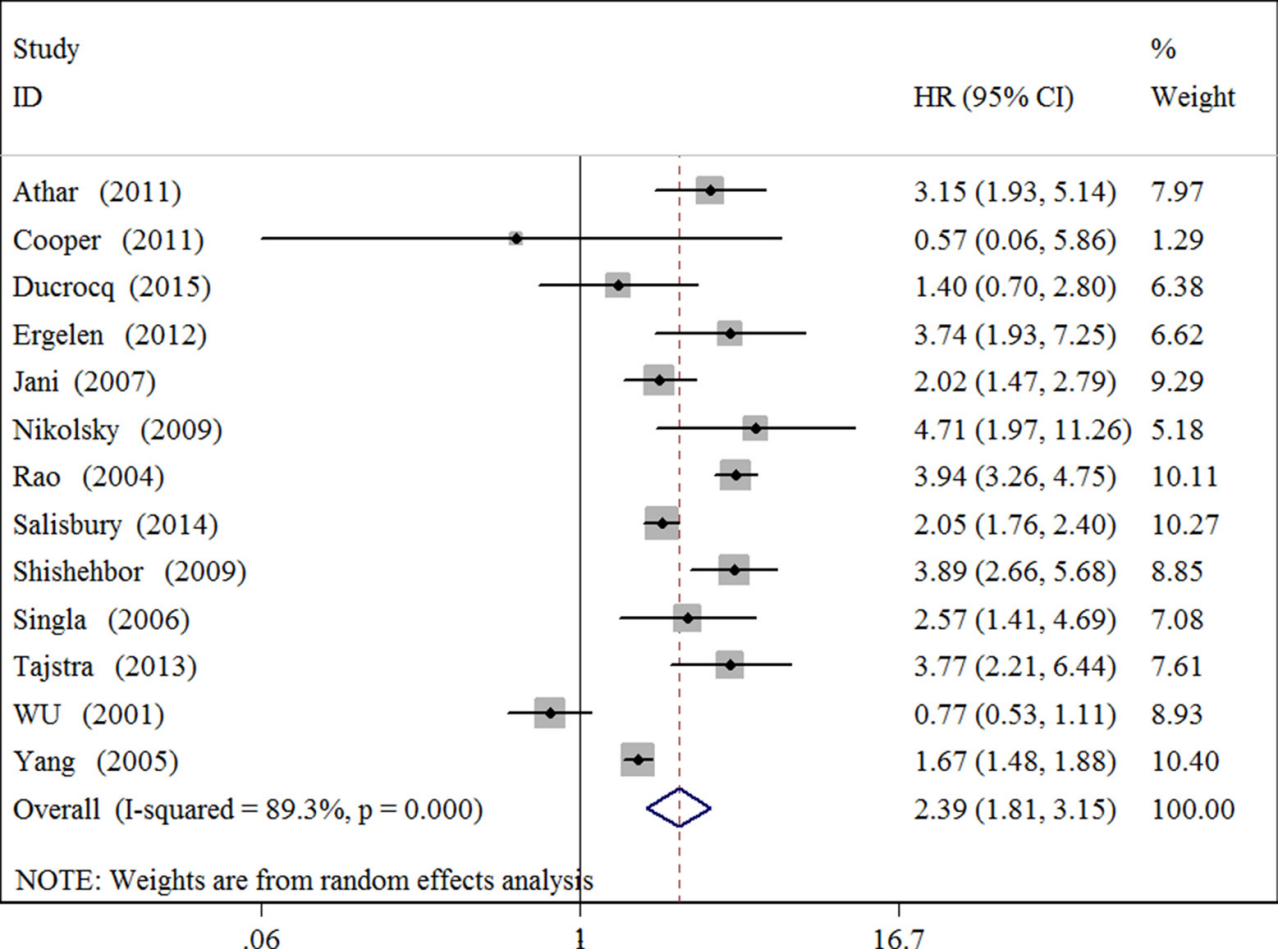
Forest plot for the association between blood transfusion and short-term all-cause mortality in patients with myocardial infarction

Thirteen cohort studies investigating blood transfusion and in hospital/30-day all-cause mortality for patients with MI were included in the analysis. The summary HR for blood transfusion versus non-blood transfusion was 2.39 (95% CI 1.81 to 3.15) (Figure 2 and Table 1A). There was evidence of significant heterogeneity among studies (I^2^ = 71.0%, *P* = 0.002). Meta-analysis also demonstrated a significant association between blood transfusion and in hospital all-cause (HR 2.21 95% CI 1.78 to 2.74) or 30-day mortality (HR 2.40 95% CI 1.22 to 4.74). When stratified by categories of study baseline characteristics including study region, center number, design, sample size, MI type, adjustment, study quality, publication type, hemoglobin level and hematocrit, compared to non-blood transfusion, blood transfusion was significantly associated with increases in mortality for almost all of these subgroups (Table 1A). These effects seems to be consistent with low heterogeneity for single center studies (I^2^ = 0), studies with large sample size (≥ 3000) (I^2^ = 34.1%), MI type of non-STEMI (I^2^ = 47.4%) and STEMI (I^2^ = 0), low hemoglobin level (≤ 12g/dl) (I^2^ = 16.9%) or high hemoglobin level (> 12g/dl) (I^2^ = 0).

**Figure 3:**
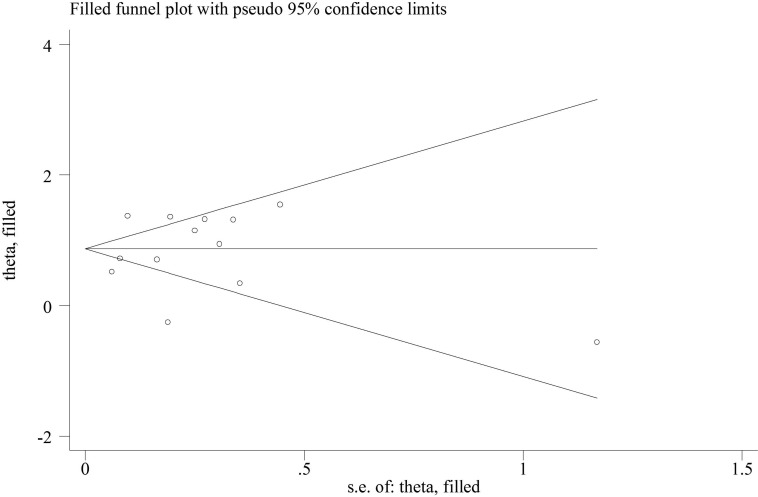
Trimmed and filled funnel plot for blood transfusion and short-term all-cause mortality in patients with myocardial infarction

Funnel plot for short-term mortality outcome indicated relatively good symmetry, with no evidence of the presence of publication bias (Figure 3). There was also no evidence of publication bias according to Begg's test (*P* = 0.855) and Egger's test (*P* = 0.511).

### Long-term all-cause mortality

Nine cohort studies investigating blood transfusion and long-term all-cause mortality for patients with MI were included in the analysis. The summary HR for blood transfusion versus non-blood transfusion was 1.90 (95% CI 1.40 to 2.58). There was significant heterogeneity among studies (I^2^ = 80.9%). Detailed subgroup analyses are presented in Table 1B. We did not test publication bias due to the limited number of included studies (< 10).

## DISCUSSION

The present updated meta-analysis of cohort studies indicated that blood transfusion therapy was associated with increases in short-term and long-term all-cause mortality for MI patients. Most stratified analyses based on clinical characteristics did not alter the associations between blood transfusion and mortality for MI patient. We also find that significant heterogeneity could partly attibute to 发factors such as number of study center, sample size, MI type and hemoglobin level.

One previous meta-analysis has returned similar results that included 10 studies reported that the increased all-cause mortality was associated with blood transfusion (versus no blood transfusion) for MI (HR, 2.91, 95% CI, 2.46 to 3.44). In the past several years, however, an increasing number of studies have reported on the association between blood transfusion and MI patient mortality. We updated our data and found consistence with the previous analysis in terms of short-term and long-term all-cause mortality which were all significantly increased in people with MI.

This updated meta-analysis has synthesised evidence from cohort studies published until 2016 on both short-term and long-term risk of mortality in MI patients. Our meta-analysis, however, included 17 cohort studies with relatively low risk of bias, which allowed us to examine the association between blood transfusion and the risk of mortality in a large dataset with a harmonised exposure and comprehensive outcome measurements (both short-term and long-term outcomes).

Several limitations of our study should also be acknowledged. Firstly, we have performed meta-analyses using aggregated patient data instead of individual patient data which would have allowed comprehensive adjustment for baseline characteristics. Moreover, three of the included studies were abstracts with limited information and potential flaws in methodological quality. However, sensitivity analysis by excluding these studies did not alter the trend of the main analysis for both short-term and long-term all-cause mortality, indicating the robustness of the meta-analysis. Secondly, we did not stratify risk of mortality of different transfused blood components due to the lack of original data from the included studies. Thirdly, unpublished literature was not searched, which might be associated with increased risk of missing some unpublished data with negative or null results. Furthermore, though heterogeneity did exist in the study population, we have abstracted characteristics of each included study, such as acute or non-acute STEMI and NSTEMI. The result showed that blood tranfusion was significant associated with increased short-term all-cause mortality in patients with STEMI (HR 3.83, 95% CI 2.89 to 5.07), non-STEMI (HR 1.83, 95% CI 1.29 to 2.70) and mixed type (HR 2.09, 95% CI 1.38 to 3.16), which was consistent with the result in the general MI patients. As presented in Table 1, the results of most of the subgroup analyses were agree with that of the primary analysis. Another limitation was that immortal time bias was likely to be present, which might affect any analysis, especially survival analyses, that applied a time-fixed definition to an exposure that actually varied over time [25, 26]. However, we tried to avoid this bias by using time-to-event data for analyses. Finally, some of the included studies were retrospective in study design, which might have brought about recall bias. Therefore, we were still unable to determine whether blood transfusion would be prognostic factor for MI. Further randomised controlled trials in those patients are required.

In patients with MI, blood transfusion treatment is associated with patient short-term and long-term all-cause mortality. However, further large-scale prospective studies based on individual patient data are needed to establish its validity of this association.

## MATERIALS AND METHODS

### Literature search

On June 5th, 2016, a systematic literature search of PUBMED, EMBASE, and the Cochrane central register of controlled trials databases was conducted using the keywords and medical subject heading (Mesh) terms: (transfusion* OR blood transfusion) AND (myocardial infarct OR cardiovascular stroke OR heart attack OR acute coronary syndrome OR heart infarction) AND (survival OR prognos* OR mortality OR predict* OR death OR fatality). We also performed a manual search of the reference lists in all selected studies based on the Preferred Reporting Items for Systematic Reviews and Meta-Analysis checklist (PRISMA). We presented the detailed search strategies of the three major databases in Supplementary Search Strategy.

### Study selection and eligibility criteria

Two investigators (Z.Y. and B.Y.) independently screened all study titles and/or abstracts and excluded non-original studies including letters, reviews, case reports, comments and studies without sufficient data for analysis. We did not include non-English-language studies due to the low study quality and insufficient data for analyses. In case of disagreement among the investigators, joint revision was performed by two senior authors (W.L. and K.L.) until consensus was achieved. As is shown in Figure 1, the flowdiagram of study selection summarizes how we identify eligible studies.

We included prospective or retrospective observational studies in any language evaluating blood transfusion on the mortality for patients with MI. Study selection was not restricted by MI types or MI onset type including acute or non-acute ST elevation myocardial infarction (STEMI) and non-ST elevation myocardial infarction (NSTEMI). Studies were excluded if the prognostic data was not reported. If multiple studies reporting on overlapping cohorts, the one with the most informative one was included. We also excluded those non-peer reviewed meeting abstracts for low methodological quality and limited information available. The corresponding authors of eligible studies would be contacted for additional data if they did not report sufficient data for meta-analysis

### Data extraction

Two investigators (Z.Y. and B.Y.) extracted data from the included studies with a predefined standardised data collection form. The detailed study and patient characteristics as well as the survival data were presented as follows: study first author, research country, patient inclusion period, single or multicenter involved, study design, sample size, male patient percentage, MI type, baseline hemoglobin level, baseline hematocrit, study outcomes, follow up period and adjusted variables.

### Study end points and quality assessment

We classified outcomes as short-term and long-term outcomes. Short-term outcomes included in-hospital all-cause mortality and 30-day all-cause mortality, which was defined as the time elapsing from the date of patient enrollment in hospital to the date of death within 30 days or before discharge from hospital irrespective of the cause of death. Long-term outcomes included all-cause mortality after 30 days from the date of patient enrollment in hospital.

Study quality was assessed by two reviewers (Z.Y. and B.Y.) independently using the Newcastle-Ottawa scale (NOS) [27] for cohort studies. The following three categories were evaluated: cohort selection (maximum 4 stars), comparability of cohorts (maximum 2 stars) and assessment of outcome (maximum 3 stars). Those that obtained a maximum of 9 stars indicated a higher quality of the study.

### Statistical analysis

The data for each study were exported to R software version 3.1.2 using the ‘metafor’ package (R Development Core Team 2013) for statistical analysis. Adjusted HRs of each study were combined to compute summary estimates using a random-effects model [28]. Statistical heterogeneity among studies was evaluated by the Cochran Q statistic and quantified with the I^2^ statistic with an I^2^ > 50 % suggesting significant heterogeneity [29]. Finally, publication bias was evaluated through visual inspection of funnel plot asymmetry as well as by the Begg's and Egger's regression tests [30, 31]. Sensitivity analyses were performed by omitting each study at one time and recalculating the others to explore whether the combined estimates altered substantially by any one of the omitted study. Sensitivity analysis was also performed by using Duval's nonparametric trim-and-fill method to assess the potential influence of publication bias [32].

## SUPPLEMENTARY MATERIALS TABLES




