# Supplementary material for: Blood transfusion and mortality in myocardial infarction: an updated meta-analysis
Source: Oncotarget. 2017 Jul 12;8(60):102254–62. doi: 10.18632/oncotarget.19208 (PMC5731951; doi:10.18632/oncotarget.19208)
Supplement: Supplementary file 1 [file oncotarget-08-102254-s001.pdf]

Blood transfusion and mortality in myocardial infarction: an updated meta-analysis

SUPPLEMENTARY MATERIALS

SUPPLEMENTARY SEARCH STRATEGY

Pubmed

1. "Myocardial Infarction"[Mesh]
2. "Acute Coronary Syndrome"[Mesh]
3. (Myocardial Infarct* OR Cardiovascular Stroke* OR Heart Attack* OR Acute Coronary Syndrome* OR heart infarction) [Title/Abstract]
4. 1 OR 2 OR 3
5. "Blood Transfusion"[Mesh]
6. Transfusion*[Title/Abstract]
7. 5 OR 6
8. "Survival"[Mesh]
9. "Prognosis"[Mesh]
10. "Mortality"[Mesh]
11. (prognos* OR survival OR mortality OR predict* OR outcome* OR death) [Title/Abstract]
12. 8 OR 9 OR 10 OR 11
13. 4 AND 7 AND 12

Embase

1. 'heart infarction'/exp
2. 'acute coronary syndrome'/exp
3. (‘Myocardial Infarct*’ OR ‘Cardiovascular Stroke*’ OR ‘Heart Attack*’ OR ‘Acute Coronary Syndrome*’ OR ‘heart infarction’)
4. 1 OR 2 OR 3
5. "blood transfusion'/exp
6. transfusion*:ab,ti
7. 5 OR 6
8. ‘mortality'/exp
9. 'survival'/exp
10. 'prognosis'/exp
11. 8 OR 9 OR 10
12. (prognos* OR survival OR mortality OR predict* OR outcome* OR death) :ab,ti
13. 11 OR 12
14. 4 AND 7 AND 13

Cochrane Library

1. MeSH descriptor: [Myocardial Infarction] explode all trees
2. MeSH descriptor: [Acute Coronary Syndrome] explode all trees
3. (Myocardial Infarct* or Cardiovascular Stroke* or Heart Attack* or Acute Coronary Syndrome* or heart infarction):ti,ab,kw (Word variations have been searched)
4. 1 OR 2 OR 3
5. MeSH descriptor: [Blood Transfusion] explode all trees
6. Transfusion*:ti,ab,kw (Word variations have been searched)
7. 5 OR 6
8. ' MeSH descriptor: [Mortality] explode all trees
9. MeSH descriptor: [Survival] explode all trees
10. MeSH descriptor: [Prognosis] explode all trees
11. (prognos* or survival or mortality or predict* or outcome* or death):ti,ab,kw (Word variations have been searched)
12. 8 OR 9 OR 10 OR 11
13. 4 AND 7 AND 12.

Supplementary Table 1: Basic characteristics of the eligible studies investigating the associations between blood transfusion and outcomes in patients with myocardial infarction. See Supplementary_ Table_1.
