# Supplementary material for: Blood transfusion and mortality in myocardial infarction: an updated meta-analysis
Source: Oncotarget. 2017 Jul 12;8(60):102254–62. doi: 10.18632/oncotarget.19208 (PMC5731951; doi:10.18632/oncotarget.19208)
Supplement: Supplementary file 2 [file oncotarget-08-102254-s002.docx]

**Supplementary Table 1:** Basic characteristics of the eligible studies investigating the associations between blood transfusion and outcomes in patients with myocardial infarction.

| **Study** | **Region** | **Inclusion period** | **Single /multicentered** | **Study design** | **Sample size (Exposure)** | **Male Sex (%)** | **MI type** | **Baseline hemoglobin level, g/dl** | **Baseline hematocrit, %** | **Outcomes** | **Follow up period** | **Adjusted variables** |
| --- | --- | --- | --- | --- | --- | --- | --- | --- | --- | --- | --- | --- |
| Aronson  (2008) | Israel | -2008 | Multi | Prospective | 2326  (192) | 1788  (76.9) | AMI | 11.8 | NR | 6-month mortality;6-month mortality | 6-month | Transfusion propensity, baseline characteristics and nadir hemoglobin |
| Athar  (2011) | US | 2003- 2007 | Multi | Retrospective | 1496  (148) | 983  (65.7) | AMI | NR | 28 | In-hospital mortality | NR | Age, sex, race, history of HTN, DM, CHF, COPD, and CVA and lowest hematocrit in the first 24 hours of CCU admission |
| Cooper  (2011) | US | 2003- 2009 | Multi | Prospective | 45  (21) | 22  (48.9) | AMI | NR | 26.9 | In-hospital mortality;30-day mortality | 30-day | Unclear |
| Cosgrove  (2009) | US | 2003- 2004 | Multi | Retrospective | 5588  (207) | NR | STEMI | NR | NR | Long-term mortality | 1000d | Age, sex, race, diabetes, hypertension, renal disease, anemia, cancer, cerebrovascular disease and left ventricular dysfunction, use of Gp2b/3a inhibitors, type of stents used, transfusion during PCI for STEMI (vs. Non transfusion) |
| Ducrocq  (2015) | France | 2005 | Multi | Prospective | 2636  (151) | 2461  (67.1) | AMI | 11.4 | NR | In-hospital mortality;5-year mortality | 5 years | Age; sex; risk factors; history of cardiovascular disease; other comorbidities; previous medications; type of myocardial infarction; Global Registry of Acute Coronary Events (GRACE) risk score; medications used in the first 48 hours of hospital admission; coronary angiography, PCI, or coronary artery bypass graft surgery during hospital stay; and in-hospital bleeding, anemia, and transfusion. |
| Ergelen  (2012) | Turkey | 2003- 2008 | Single | Retrospective | 2644  (88) | 2111  (79.8) | STEMI | 11.1 | 33 | Long-term mortality | 21 months | Killip class 2, 3 at admission, RBC transfusion, DM, and renal failure |
| Jani  (2007) | US | 1997- 2004 | Multi | Prospective | 4623  (1031) | 2667  (57.7) | AMI | 10.47 | NR | In-hospital mortality | NR | Adjustment for comorbidities and propensity for transfusion, |
| Jolicœur  (2009) | US | 2004- 2006 | Multi | Prospective | 5532  (213) | 4250  (76.8) | STEMI | 12.9 | NR | 90-day mortality | 90-day | Age, sex, race, weight, history of diabetes, systolic blood pressure and heart rate, total ST-segment change, qualifying Killip class, anterior myocardial infarction, intra-aortic balloon pump, enrolment in North America, baseline hemoglobin, creatinine clearance, diastolic blood pressure, heart rate, prior CABG, diabetes, use of beta-blockers, nitrate |
| Nikolsky  (2009) | US | -2009 | Multi | Prospective | 2060  (82) | 1505  (73.1) | AMI | 13.1 | 38.7 | 30-day mortality;1-year mortality | 1-year | Baseline anemia and transfusion propensity |
| Rao  (2004) | US | -2004 | Multi | Prospective | 24112  (2401) | 15821  (65.6) | ACS | NR | 39.9 | 30-day mortality | 30-day | Age, race, weight in kilograms, US vs non-US site, diabetes mellitus, systolic blood pressure, diastolic blood pressure, heart rate at baseline, time from symptom onset to hospitalization, prior stroke, prior MI, sex, history of angina prior to qualifying episode, hypertension, hyperlipidemia, family history of coronary artery disease, history of congestive heart failure, peripheral vascular disease, prior percutaneous coronary intervention,, Killip class, baseline hematocrit, maximum creatine kinase ratio at baseline, chronic renal insufficiency, ST-segment elevation or depression on initial electrocardiogram, beta-blocker use at baseline, calcium channel blocker use at baseline, nitrate use at baseline, and current smoking, Bleeding and Transfusion Propensity, and Nadir Hematocrit |
| Salisbury  (2014) | US | 2000- 2008 | Multi | Prospective | 34937  (1778 ) | 20203  (57.8) | AMI | 9.7 | NR | In-hospital mortality | NR | Case-mix differences |
| Shishehbor  (2009) | US | -2009 | Multi | Prospective | 4131  (307) | 2723  (76.2) | STEMI | 13.9 | 41.4 | 30-day mortality, 1-year mortality | 1-year | Age; gender; race; height; weight; country of origin; comorbidities including diabetes, hypertension, hypercholesterolemia; smoking; chronic obstructive pulmonary disease; chronic renal insufficiency; peripheral arterial disease; heart failure; stroke; cancer diagnosed in the past 5 years; and history of coronary artery bypass grafting and percutaneous coronary artery interventions. |
| Singla  (2006) | US | 2001 | Single | Prospective | 1410  (110) | 361  (97.6) | ACS/NSTEMI | 8.91 | NR | 30-day mortality | 30-day | Hypotension on presentation, pulmonary edema, and increased troponin-I levels |
| Tajstra  (2013) | NR | 1999- 2004 | NR | Prospective | 2415  (82) | NR | STEMI | NR | NR | In-hospital mortality;5-year mortality | 5-year | Baseline differences, RBC transfusion |
| Volenti  (2010) | NR | 1995- 2007 | NR | Retrospective | 2771  (93) | 2066  (74.6) | AMI | 8.1 | NR | 6-month mortality | 6-month | Adjusting for the propensity-score |
| Wu  (2001) | US | 1994- 1995 | Multi | Retrospective | 78974  (3680) | 36249  (45.9) | AMI | NR | Graded levels | 30-day mortality | 30-day | The score on the Acute Physiology and Chronic Health Evaluation (APACHE II), the presence or absence of do not-resuscitate order on admission, the location of the myocardial infarction, the presence or absence of congestive heart failure, the mean arterial pressure, the heart rate, and the presence or absence of renal insufficiency, use or nonuse of primary reperfusion therapy, the use or nonuse of aspirin on admission, the use or nonuse of beta-blockers on admission, and predictors of the use of blood transfusion. |
| Yang(2005) | US | 2001- 2004 | Multi | Retrospective | 85111  (12724) | 50742  (59.2) | ACS/NSTEMI | NR | 35 | In-hospital mortality;30-day mortality | 30-day | CRUSADE clinical model. |

Abbreviations: ACS, acute coronary syndrome; AMI, acute myocardial infarction; CCU, coronary care unit; COPD, chronic obstructive pulmonary disease; CHF, congestive heart failure; DM, diabetes mellitus; MI, myocardial infarction; Multi, ,multicentered; NR, not reported; NSTEMI, non- ST segment elevation myocardial infarction; PCI, percutaneous coronary intervention; STEMI, ST segment elevation myocardial infarction.
